# Isotopic Nitrogen-15 Labeling of Mice Identified Long-lived Proteins of the Renal Basement Membranes

**DOI:** 10.1038/s41598-020-62348-6

**Published:** 2020-03-24

**Authors:** Pan Liu, Xinfang Xie, Jing Jin

**Affiliations:** 1 Feinberg Cardiovascular and Renal Research Institute, Feinberg School of Medicine, Northwestern University, Chicago, IL 60611 USA; 2 Department of Nephrology, The First Affiliated Hospital of Medical College, Xi’an Jiaotong University, Xi’an, China

**Keywords:** Biochemistry, Biological techniques

## Abstract

The kidney is comprised of highly complex structures that rely on self-maintenance for their functions, and tissue repair and regeneration in renal diseases. We devised a proteomics assay to measure the turnover of individual proteins in mouse kidney. Mice were metabolically labeled with a specially formulated chow containing nitrogen-15 (^15^N) with the absence of normal ^14^N atoms. Newly synthesized proteins with ^15^N contents were distinguished from their ^14^N counterparts by mass spectrometry. In total, we identified over 4,000 proteins from the renal cortex with a majority of them contained only ^15^N. About 100 proteins had both ^14^N- and ^15^N-contents. Notably, the long-lived proteins that had large ^14^N/^15^N ratios were mostly matrix proteins. These included proteins such as type IV and type VI collagen, laminin, nidogen and perlecan/HSPG2 that constitute the axial core of the glomerular basement membrane (GBM). In contrast, the surface lamina rara proteins such as agrin and integrin had much shorter longevity, suggesting their faster regeneration cycle. The data illustrated matrix proteins that constitute the basement membranes in the renal cortex are constantly renewed in an ordered fashion. In perspective, the global profile of protein turnover is usefully in understanding the protein-basis of GBM maintenance and repair.

## Introduction

The basement membrane matrices have important roles in the kidney. The glomerular basement membrane (GBM) is an integral part of the filtration barrier between the layers of fenestrated endothelial cells and the interdigitated podocyte foot processes^1,2^. Characterized as a finely interwoven network of collagen and laminin fibers, its long-term integrity is maintained by the adjacent cells through an incompletely understood process^3,4^. It is known that the matrix proteins are produced, and presumably also recycled, by these two cell types. Genetic mutations of the genes encoding the GBM proteins are associated with kidney diseases, such as Alport syndrome and Pierson syndrome^5–12^. In a number of kidney diseases caused by systemic conditions such as diabetes, autoimmune including Goodpasture disease, membranous nephropathy and dense deposit disease of C3 glomerulopathy, among others, thickening and irregularity of the GBM are the characteristic features of the respective diseases^13–16^. Although the mechanisms for the pathological transformation remain unknown, it can be postulated that the normal turnover needed for maintaining the balance of matrix proteins is altered.

In order to gain insight into the natural dynamics of protein turnover in the kidney, particularly in extracellular matrices, we conducted a proteomic study following 12 weeks of feeding mice with stable isotope nitrogen-15 (^15^N). During this period of time, newly synthesized proteins had incorporated ^15^N in their proteins. By performing mass spectrometry to compare the ^14^N versus ^15^N content of each protein through a proteomic method known as stable isotope labeling of mammals (SILAM)^17,18^, we measured the turnover dynamics of thousands of proteins in the mouse kidney.

## Results

### Extremely long-lived proteins in the urea-insoluble fraction of kidney lysate

**Figure 1:**
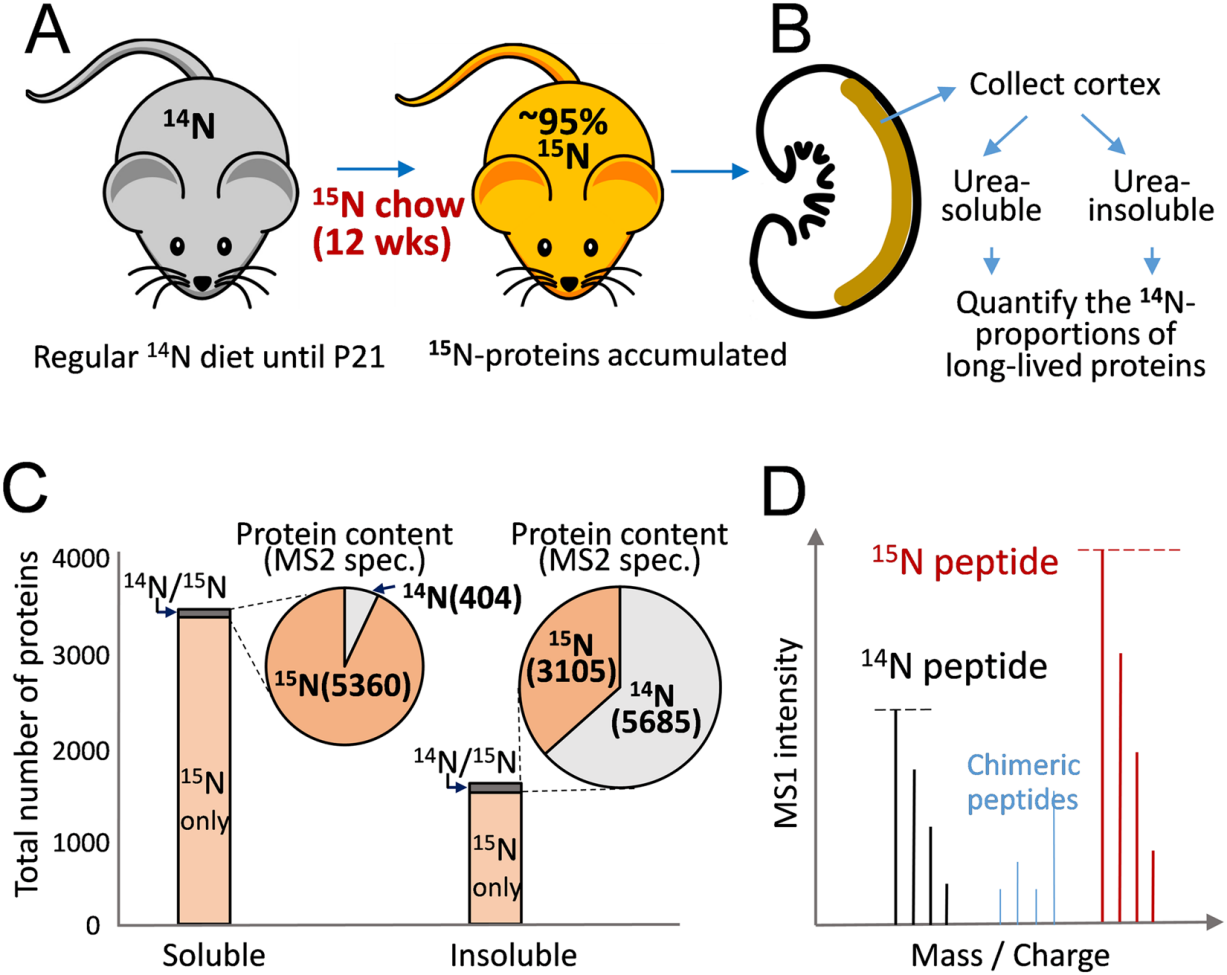
The ^15^N labeling workflow in conjunction with mass spectrometry identified long-lived proteins in the kidney cortex. (**A**) C57BL/6 J mice were subjected to an exclusive ^15^N chow starting on P21 for 12 consecutive weeks. ^15^N was incorporated into newly synthesized proteins (represented by red color). (**B**) Three kidneys from three mice were used to collect the cortex tissues (marked with brown color), which were then combined and extracted for proteins using 8 M urea solution. The samples were fractionated into urea-soluble and urea-insoluble fractions and separately digested with Trypsin/Lys-C endopeptidases. The resulting peptides were detected as either fully ^14^N- or fully ^15^N-peptides by LC-MS/MS and the remaining ^14^N-content of each individual protein as the proportion of the total protein amount was calculated. (**C**) In the bar graph, the total number of individual proteins detected either through their ^14^N- or ^15^N-peptides were counted. In total, 3482 individual proteins were detected from the urea-soluble fraction and 1562 from the urea-insoluble fraction, of which the majority proteins with the exceptions of 87 and 109 proteins respectively were identified only by their labeled ^15^N peptides. As shown in the pie-chart insets, between the 87 and 109 protein groups we evaluated their ^14^N versus ^15^N contents as a whole based on the sum of MS2 spectral counts of all proteins in each group (value in parenthesis). Clearly, the soluble fraction contained more than 10 times less remaining ^14^N proteins in total quantity, 404 versus 5685 spectra. (**D**) In order to calculate the proportions between ^14^N and ^15^N contents of an individual protein, we relied on the MS1 intensity values of its peptide spectra (represented by the black and red spikes respectively), and collectively calculated the composite ratios of respective under-curve-areas based on the reconstructed chromatographs of corresponding ^14^N versus ^15^N peptide pairs (not shown, details in Methods). Chimeric peptides shown in blue represent partially labeled peptides that contained both ^14^N and ^15^N, which were not accounted for in the calculation of ^14^N proportion of individual proteins. The figures were created in Adobe Illustrator v24.0 (https://www.adobe.com/products/illustrator.html).

Individual proteins have a wide range of *in vivo* turnover rate^19,20^, and the balance between protein synthesis and catabolism is tightly regulated in health and disease conditions. As we intended to address the long-standing question as to whether mature GBM is actively renewed^4,21^, we had chosen a 12-week labeling schedule. Starting from postnatal age of P21 for a consecutive 12 weeks, C57BL/6 J mice were subjected to dietary treatment of exclusive blue-green algae-based chow with all nitrogen atoms being ^15^N with the absence of ^14^N (Fig. 1A and Methods). These animals developed normally^22^. From the kidney cortex, we extracted most cellular proteins with 8 M urea solution, whereas matrix proteins were enriched in the urea-insoluble fraction (Fig. 1B). By performing liquid chromatography-tandem mass spectrometry (LC-MS/MS), 3482 proteins were identified in the urea-soluble fraction and 1562 proteins in the urea-insoluble fraction (Fig. 1C). Among these proteins, only 87 and 109 proteins in the soluble and insoluble fractions respectively were also detectable with their ^14^N-peptides that had existed for 12 weeks or longer. When the relative amounts between older ^14^N and newer ^15^N proteins were evaluated by MS2 spectral count values, the insoluble fraction contains a larger quantity of the older proteins than the soluble fraction (measured by total spectral count: 5685 vs. 404 as in Fig. 1C insets: pie charts), suggesting matrix proteins in general live longer.

### Long-lived collagen and laminin of the glomerular basement membrane

Beside the proteins that had been completely replaced by their new ^15^N counterparts, those detected with both ^14^N- and ^15^N-peptides had let us estimate their old versus new proportions based on MS1 signal intensities of the corresponding ^14^N and ^15^N spectral pairs (Fig. 1D). We note that proteins with high abundance of remaining ^14^N were the extracellular matrix proteins and certain histone protein isoforms (Supplementary File 1). The longevity of histone isoforms such as H3.1 and H3.2, and some H2 variants were likely due to their expression associated with DNA replication, and that they are not degraded as cells divide. Under these circumstances, their abundance in ^14^N contents suggests these histone variants were mainly from slow- or non-dividing cells in the kidney.

**Figure 2:**
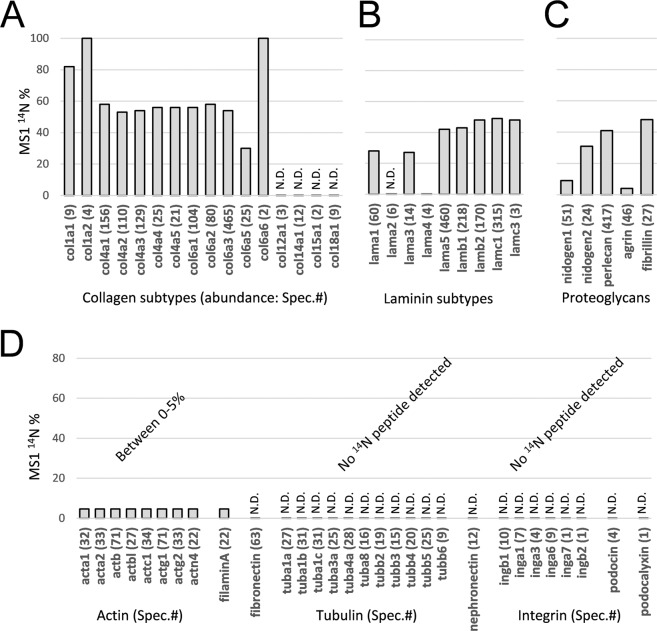
Collagen, laminin and other components of the GBM have the most long-lived proteins with high remaining ^14^N contents. (**A–C**) The matrix proteins such as collagen, laminin and proteoglycan proteins were analyzed for their remaining ^14^N proportions in the urea-insoluble fraction. The *y*-axis is the percentage of ^14^N content and the *x*-axis lists all identified proteins in the group. (**D**) In contrast, intracellular proteins had more complete turnover to ^15^N. Actin, tubulin cytoskeletal proteins and membrane proteins connecting to the GBM such as integrin and podocin all had low (between 0–5%) or no ^14^N detected (N.D.: not detected). Note that artificial upper and lower limits of the ^14^N/^15^N ratios were set at 20 and 0.05, and therefore any measured ^14^N contents between 0–5% had been artificially roundup to 5% as in actin and filamin-A.

Matrix proteins such as type IV and VI Collagens, Laminins and Perlecan (basement membrane-specific heparan sulfate proteoglycan/HSPG/HSPG2) all had high ^14^N contents. In total, sixteen distinct collagen isoforms were detected. Collagen-XII, XIV, XV and XVIII were only detected in the fully ^15^N channel, indicating complete turnover. Meanwhile, collagen-I, IV and VI, which abundance levels were higher, retained 30–83% ^14^N in the urea-insoluble sample (Fig. 2A). Collagen 4α1–5 that are the main components of the renal basement membrane structures were detected at varying levels, from 21 spectra for Collagen 4α5 to 156 spectra for Collagen 4α1 (Supplementary File 1), however, all had similar ^14^N contents between 54% and 59% that reflected their individual turnover dynamics (Fig. 2A). Collagen 4α1 and 4α2 are components of the tubular basement membrane (TBM) and Collagen 4α3–5 form the key fibrous meshwork of the GBM. Their maintenance, regeneration and remodeling have been extensively studied in normal kidney physiology as well as renal diseases, such as X-linked and autosomal recessive Alport syndrome carrying genetic mutations in COL4A3, COL4A4 or COL4A5 gene.

Similarly to collagen, laminin retained 24–49% of their ^14^N contents across their nine identified isoforms of α1–5, β1 and 2, and γ1 and 3 (Fig. 2B)(Mutations of LAMB2 that encodes Laminin β2 are associated with Pierson syndrome). As it appeared, only Laminin α5, β1, β2 and γ1 were detected at high levels (Fig. 2B), suggesting α5/β1/γ1 (referred to as LM-511) and α5/β2/γ1 (LM-521) being the dominant configurations of the triple helices. Among them 42% to 49% of remaining ^14^N contents detected, which was slightly lower than of percentages of type IV collagen, possibly indicating faster turnover of laminin α5/β1,2/γ1 than collagen IV.

With respect to the linker proteoglycan proteins of the GBM meshwork, namely nidogen and perlecan that bind collagen-IV, as well as nidogen and agrin that bind the laminin matrix^23^, they had varying percentage contents of ^14^N (Fig. 2C). While Perlecan and Nidogen-2 (also known as osteonidogen) had 41% and 31% ^14^N remaining with 417 and 24 total spectra identified respectively, Agrin and Nidogen-1 (also known as entactin) had only 4.7% and 9% ^14^N remaining represented by 46 and 51 total spectra respectively. It should be noted that Agrin and Nidogen-2 form the lamina rara between the GBM and the cell layers^24,25^ that are expected to turnover more completely than the GBM core proteins. Therefore it was unexpected that Nidogen-2 out-lived Nidogen-1. Transiently expressed ECM protein such as nephronectin (Npnt) had reached complete turnover, as well as fibronectin that forms direct contact with cell surface proteins (Fig. 2D). Moving further towards the cell layers, transmembrane proteins such as integrin and podycalyxin that anchor the cells to the GBM had all reached their complete turnover from ^14^N to ^15^N (Fig. 2D). Also as expected, intracellular proteins such as actin, tubulin, podocin were completely or almost completely reconstituted with the newer ^15^N atoms (Fig. 2D).

### Urea-insoluble GBM proteins lived longer than their soluble counterparts – suggesting the surface layers more frequently renewed

**Figure 3:**
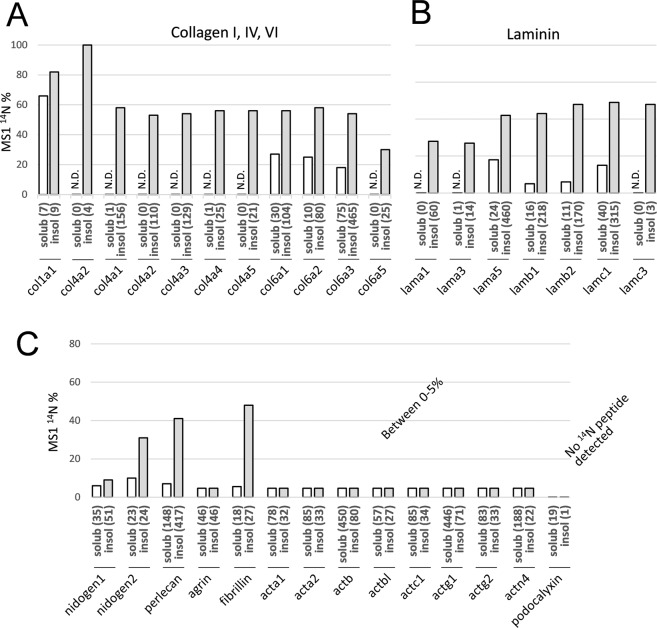
Comparison of remaining ^14^N percentage among individual proteins in urea-soluble versus insoluble fractions. (**A**,**B**) Individual collagen and laminin proteins with their total levels (value as MS2 spectral count in parenthesis) and remaining ^14^N percentage (*y*-axis value) separately compared between their urea-soluble (open bar) and insoluble isolates (filled bar). Note that proteins in the soluble fraction had either no or less ^14^N proportions than their insoluble counterparts. (**C**) Other GBM proteins such as nidogen, perlecan and fibrillin all had the similar profile, whereas cytoskeletal actin, although more abundantly harvested in the soluble fraction (spectral count value in parenthesis), with its insoluble contents completely turned over to ^15^N.

Although extraction with 8 M urea was a crude method to fractionate cellular versus matrix proteins, at a global level, it provided an approximate estimation of how tightly each protein was associated to the matrices based on solubility. In this regard, for any individual proteins as being expressed by cells and integrated into matrix, we sought to explore whether they initially adopted a relatively “loose” association with the matrix (likely through non-covalent binding that did not withstand 8 M urea). To this end, we directly compared the ^14^N proportions of the same protein in urea-soluble versus insoluble fractions (Fig. 3A).

Remarkably, collagen-IV was nearly absent with only 2 spectra recorded in the soluble fraction, as compared to 441 spectral counts recorded in the insoluble fraction, suggesting covalent association of collagen-IV to the matrix immediately after being assembled into the GBM. As for soluble laminin, there were 24, 16, 11 and 40 spectra recorded for α5, β1, β2 and γ1 respectively. The ^14^N percentages were 18%, 5%, 6% and 15%, respectively, in contrast to 42%, 43%, 48% and 49% in urea-insoluble fraction (Fig. 3B). These data indicate that newly expressed (^15^N) and assembled laminin was more loosely associated with the matrix core. Similarly, soluble perlecan had 7.4% remaining ^14^N compared to 41% ^14^N in the insoluble form; and soluble nidogen had 6% and 10% (for isoforms 1 and 2) remaining ^14^N compared to 9% and 31% of the same proteins in the insoluble fractions. By contrast, agrin had only 4.7% remaining ^14^N in both fractions (Fig. 3C), consistent with its association with the lamina rara that is constantly renewed by the podocytes and endothelia^23,25,26^.

## Discussion

By metabolic labeling of whole mice with exclusively heavy ^15^N diet for 12 consecutive weeks, we measured the remaining proportions of the ^14^N-proteins in the kidney by mass spectrometry. Most ^14^N contents were in the urea-insoluble fraction of mainly matrix proteins. It should be noted that our choice of labeling between 3 and 15 weeks was mainly based on the weaning schedule, and that ^15^N was provided conveniently at the transition to solid food. This choice of timeline would have complicated the outcome of ^15^N/^14^N ratios in a number of ways beyond regular protein turnover. For instance, when the pups grew in physical size, newer ^15^N proteins in the kidney might have swamped their ^14^N counterparts in quantity, and the calculated ratios no longer accurately reflect the rate of turnover. In addition, proteins that were not expressed before 3 weeks of age might have been inadvertently attributed to rapid turnover. Therefore, depending on the specific purpose of a study, we stress the need to design labeling schedule accordingly.

Although our global analysis does not distinguish glomerular versus tubular basement membrane matrices, known components of the GBM generally had higher proportions of long-lived ^14^N proteins. As collagen-IV α3/4/5 form the key fibrous meshwork of the GBM^9^, their maintenance, regeneration and remodeling have been extensively studied in the context of Alport syndrome caused by mutations of Collagen IV proteins of the GBM^5,7,9^. Collagen IV and VI had the highest ^14^N percentage of over 50%, followed by laminin that together forms the core structure of the GBM, and then by linker proteoglycan agrin in proximity to the cell layers^27,28^. Lastly, the cell surface proteins that interact with the GBM such as integrin all had no remaining ^14^N. In general, these percentage values are reversely correlated with the turnover rates of these proteins, which to a certain degree reflect the natural course of GBM renewal. As expected, our protein longevity results correlate with the locations of these proteins within the GBM, with collagen IV and laminin concentrated in the lamina densa, agrin enriched in the outer layer of lamina rara, and integrin forming the anchors for the adjacent cells^27^. However, it is important to note that perlecan had a much longer lifetime as compared to agrin, and similarly, linker proteins Nidogen-1 and -2 also had contrasting levels of ^14^N percentages, at 9% and 31% respectively. Our data from measuring protein turnover strongly suggest the GBM meshwork is renewed in an ordered fashion.

Overall, the study validated the general workflow of measuring molecular turnover in the renal cortex, particularly among matrix proteins that form important basement membrane structures in the kidney. It can be postulated that in human diseases that affect the histologic appearance of the GBM, such as GBM thickening and irregularity in diabetic nephropathy, ribbon-like GBM in dense-deposit disease and other immune-mediated membranous nephropathy types, there involve an altered pattern among the matrix proteins in their turnover kinetics. Testing these disease conditions with the ^15^N-labeling workflow may yield molecular insights into the underlying etiology and pathophysiology.

## Materials and Methods

### Stable isotope labeling in mouse (SILAM)

The method of ^15^N label of mice was described previously^17,22^. In brief, starting at postnatal day 21 for a consecutive 12 weeks, C57BL/6 J mice were fed exclusively with a ^15^N-raised spirulina diet (from Cambridge Isotopes and Harlan Laboratories). At the end, the ^15^N-proteins in the serum was determined to be greater than 99%^22^. All animal procedures were approved by Institutional Animal Care and Use Committee of the Northwestern University (approved protocol number IS00000429 and IS00000862), and carried out in accordance with the guidelines and regulations for the Care and Use of Laboratory Animals.

### Lysis and fractionation of the kidney tissue

Freshly collected kidneys were flash-frozen and then stored in liquid nitrogen. The tissue was harvested by surgical dissection of the renal cortex with a small portion of the outer medulla, avoiding the papilla and the renal sinus. The collected tissue was minced into pieces with a scalpel and then submerged into 4x volume of 8 M urea solution. The mixture was homogenized by sonication, followed by centrifugation at 18,000 g for 10 min. The supernatant was collected as the urea-soluble fraction. The resulting pellet was washed three times with 500 μL of 8 M urea and subjected to repeated sonication followed by centrifugation in each round. At the end, SDS sample buffer containing 4% SDS, 20% glycerol, 10% 2-mercaptoethanol, 0.004% bromphenol blue and 0.125 M Tris HCl, pH = 6.8 was added to the final pellet. After an additional sonication round, there was no visible pellet remaining and the solution was considered the urea-insoluble fraction.

### Proteomic preparation

The urea-soluble fraction was subjected to a standard in-solution digestion protocol. In brief, the 8 M solution that contained the soluble lysate was first diluted 4 times to a final solution containing 2 M urea. The proteins were subjected to standard reduction (with TCEP), alkylation (with iodoacetamide) before digested with Trypsin/Lys-C Mix (purchased from Promega Corp.) following the manufacturer’s standard digestion protocol for overnight at 37 °C. On the next day, the solution was loaded to a C18 column (Millipore) and tryptic peptides were recovered in acetyl nitrate (ACN) and then dried by vacuum. Separately, the urea insoluble fraction in SDS sample buffer was resolved by running 12% SDS PAGE. The sample was allowed to run ~1 cm into the gel, then the gel was stained with GelCode Blue (Thermo Fisher Scientific). The stained gel lane was excised into ~1 mm^3^-sized gel cubes. These gel cubes were subsequently subjected to a standard in-gel reduction, alkylation and Trypsin/Lys-C digestion (Promega) procedure.

### Mass spectrometry

The procedures for ^15^N-based proteomics were described previously^22,29^. Briefly, after trypsin digestion 3 μg of the peptides was dissolved in 94.785% H_2_O, 5% acetonitrile (ACN), 0.125% formic acid (FA) solution, and then loaded onto a nanoViper C18 trap column. Following a two hour gradient to 99.875% ACN and 0.125% FA solution the peptides were separated, and then electrosprayed from the Nanospray Flex Ion Source and analyzed on the Orbitrap Fusion Tribrid mass spectrometer. MS parameters were as follows: ion transfer tube temp was 300 °C with Easy-IC internal mass calibration, and the default charge state was 2. Detector type was set to Orbitrap at 60 K resolution, wide quad isolation, normal scan mass range between 300 and 1500 m/z. Max injection time was 50 ms, AGC target was at 200,000, microscans was at 1, S-lens RF level was 60. Without source fragmentation, datatype for positive and centroid and MIPS was on, included charge states of 2–6 (reject unassigned). Dynamic exclusion enabled with n = 1. Precursor selection decision was based on the most intense, top 20, isolation window = 1.6, scan range = auto normal, first mass = 110, collision energy 30%, CID, Detector type = ion trap, max injection time = 75 ms, AGC target – 10,000, inject ions for all available parallelizable time.

### Spectra analysis and protein quantification

Spectra analysis was done using Integrated Proteomics Pipeline (IP2), including running ProLuCID searches against the RefSeq mouse dataset. Basic parameters of 50 ppm precursor mass tolerance and 600 ppm for fragmented ions were used. Searches were filtered with DTAselect containing one peptide per protein, at least one tryptic end and unlimited missed cleavages of a minimum of 6 amino acid, with a false discovery rate (FDR) <0.001, fixed modification of +57.02146 Da on cysteine residues, and all precursor mass within 10 ppm of expected. To estimate peptide FDRs accurately (set at <1%), target/decoy database was used containing the reversed sequences of all the proteins appended to the target database^30^. Searches were done for combined light and heavy peptides and Census quantified^31^. Only fully ^14^N- or ^15^N-containing peptides were considered.

To calculate the ^14^N/^15^N peptide ion intensity, the ProLuCID results were used to reconstruct MS1 ion chromatograms in the m/z range that included both the heavy and light peptide^22,31^. The intensity ratios were then calculated per peptide using the reconstructed chromatogram. Census also allows for filtration of poor-quality peptide ratio measurements. With QuantCompare, the final peptide ratios were generated. For each protein, its heavy versus light ratios were represented by the composite of all peptide ratios identified by MS that are assigned to the protein. In cases of extremely low signals in one of the two channels, which will render extremely high or low ratio values mathematically, we arbitrarily set lower limit of the protein ratios at 0.05. The final list of RefSeq protein entries were searched against the UniProtKB database to obtain a non-redundant set of proteins based on their unique gene identifiers (listed in Supplementary File 1).

## Supplementary information

Supplementary information.

Supplementary information 2.
