# Supplementary material for: Isotopic Nitrogen-15 Labeling of Mice Identified Long-lived Proteins of the Renal Basement Membranes
Source: Sci Rep. 2020 Mar 24;10:5317. doi: 10.1038/s41598-020-62348-6 (PMC7093503; doi:10.1038/s41598-020-62348-6)
Supplement: Supplementary file 1 — Supplementary information. [file 41598_2020_62348_MOESM1_ESM.docx]

**Supplementary Information**

**Isotopic Nitrogen-15 Labeling of Mice Identified Long-lived Proteins of the Renal Basement Membranes**

Pan Liu^1^, Xinfang Xie^1,2^ and Jing Jin^1^*

^1^ Feinberg Cardiovascular and Renal Research Institute, Feinberg School of Medicine, Northwestern University, Chicago, IL 60611

^2^Department of Nephrology, The First Affiliated Hospital of Medical College, Xi’an Jiaotong University, Xi’an, China

* For correspondence: [jing.jin@northwestern.edu](mailto:jing.jin@northwestern.edu) (J.J.)

**Supplementary File 1. A complete list of urea-soluble and urea-insoluble proteins identified by mass spectrometry, including ^14^N/^15^N ratios and relative total protein amount (calculated as MS2 spectral count). The proteins were ranked by the ^14^N spectral count values.**
